# Solitary adrenal metastasis from invasive ductal breast cancer: an uncommon finding

**DOI:** 10.1186/1477-7819-8-7

**Published:** 2010-01-28

**Authors:** Xiao-Jiao Liu, Peng Shen, Xin-Feng Wang, Ke Sun, Fei-Fei Sun

**Affiliations:** 1 Department of Breast Surgery, the First Affiliated Hospital, College of Medicine, Zhejiang University, 79 Qingchun Road, Hangzhou, Zhejiang, 310003, PR China; 2 Department of Medical Oncology, the First Affiliated Hospital, College of Medicine, Zhejiang University, 79 Qingchun Road, Hangzhou, Zhejiang, 310003, PR China; 3 Department of Pathology, the First Affiliated Hospital, College of Medicine, Zhejiang University, 79 Qingchun Road, Hangzhou, Zhejiang, 310003, PR China

## Abstract

**Background:**

Invasive ductal carcinoma (IDC) of the breast usually metastasizes to the lungs, liver, bones and brain. Solitary adrenal metastasis is extremely rare. Due to the rarity of this condition, the optimal treatment is unclear. We report the first case of IDC of the breast metastasizing solely to the adrenal gland after a modified radical mastectomy but having a long-term disease-free survival while treated merely by a left adrenalectomy.

**Case presentation:**

A 64-year-old woman was found a left adrenal mass on a follow- up visit two years after taking a right modified radical mastectomy for the breast cancer. She was subsequently given a left adrenalectomy. Postoperative histopathology findings were compatible with invasive ductal carcinoma (IDC) of the breast. Due to the patient's refusal, no further treatments were offered after the adrenalectomy. The patient now is still alive and has no sign of relapse. Survival time after taking the right modified radical mastectomy and the left adrenalectomy is more than five years and three years, respectively.

**Conclusion:**

This is the first case of a patient with solitary, metachronous adrenal metastasis from IDC of the breast to be reported. For patients in this condition, complete removal of metastasized organ may translate into survival benefit.

## Background

Invasive ductal carcinoma (IDC) is the most common type of the breast cancer, which has been reported to constitute approximately 70-85% of all invasive breast carcinomas[1]. Usually, IDC can metastasize to the lungs, liver, bones and brain, but rarely to the adrenal glands[2,3]. In a study of metastatic patterns of breast cancer, Borst MJ[2] reported that in a group of the 2246 patients with IDC, none of them had shown adrenal metastasis. In fact, adrenal metastasis of breast cancer is generally associated with infiltrating lobular carcinomas (ILC) and often accompanied by synchronous multiorgan metastases[3]. A metachronous, isolated adrenal metastasis from ILC is rare, which is even rarer when it derives from IDC of the breast. So far there has been only one case of isolated adrenal metastasis arising from ILC of the breast documented [4], but the IDC with solitary adrenal metastasis has never been reported in the literature.

Due to the rarity of solitary adrenal metastasis from breast cancer, the optimal treatment is still unclear. Generally, distant visceral metastasis is an upset aspect for cancer patient, palliative chemotherapy would be recommended. However, studies on some malignant diseases [5-8] suggested that when metastasis is isolated to the adrenal gland, adrenalectomy can lead to survival benefit. Here we report the first case of IDC of the breast metastasizing solely to the adrenal gland after a modified radical mastectomy but having a long-term disease-free survival treated merely by a left adrenalectomy.

## Case presentation

**Figure 1:**
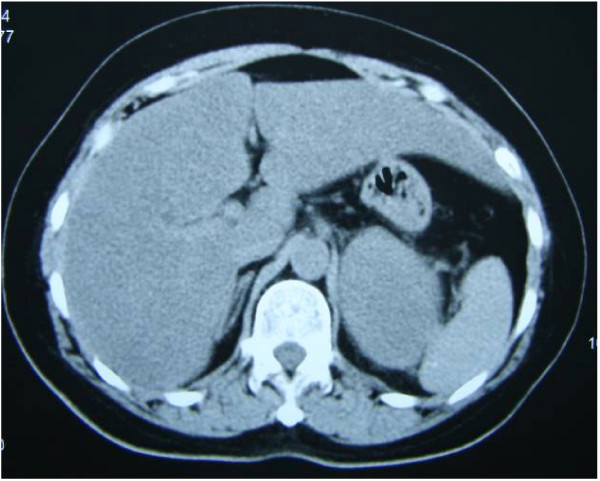
**Unenhanced CT scan showing a 5.4 × 7.0 cm, homogenous, low-density (27 HU) mass of the left adrenal**.

**Figure 2:**
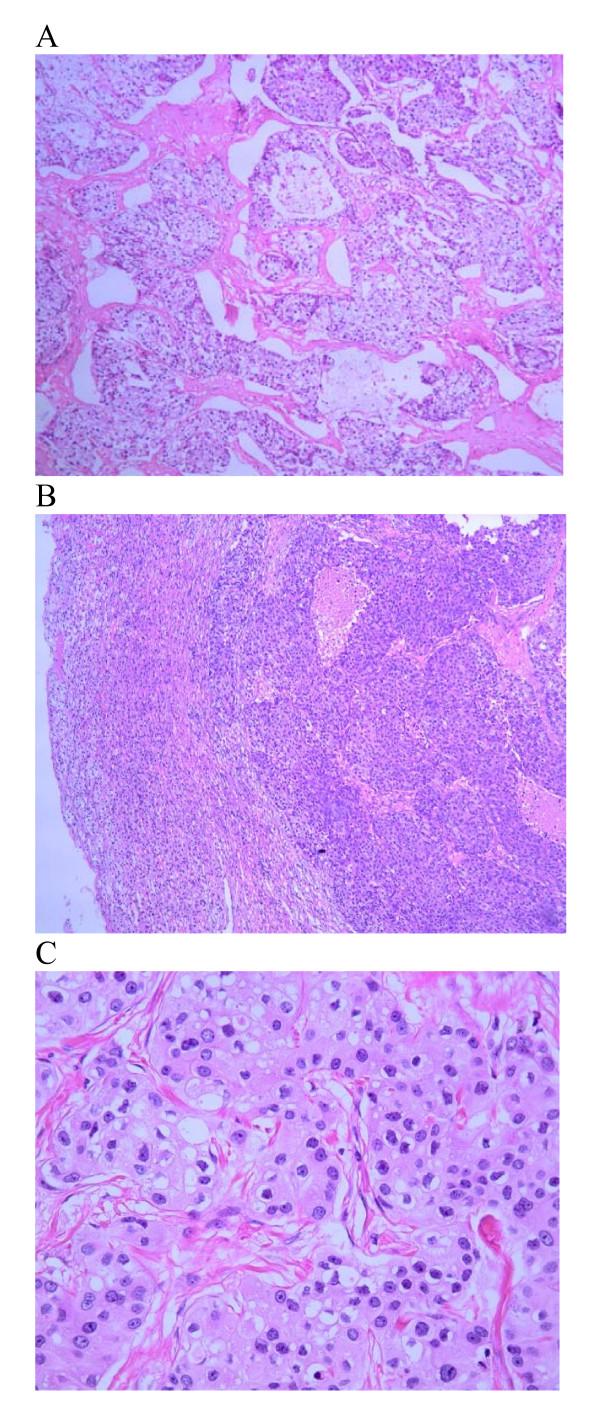
**Histological section of the primary IDC of the right breast (2A) and the adrenal metastatic disease(2B, 2C)**. The tumor cells are arranged in solid nests or cords with infiltrative growth pattern in the primary IDC (2A; H & E 10 ×), which has also been shown in the adrenal metastatic lesion (2B; H & E 10 ×) with vaying size of oval cells showing eosinophilic cytoplasm and prominent small nucleoli (2C; H & E 40 ×).

In September 2006, a 64-year-old woman was hospitalized for a left adrenal mass which was detected by a follow up visit. Ultrasonography showed a 5.4 × 7.0 cm mass on the left adrenal gland, which was confirmed by unenhanced CT scan a size of 5.4 × 7.0 cm well-shaped, homogenous, and low-density (27 HU) tumor (Figure 1). The patient was asymptomatic, and had a medical history of right breast cancer, which had been treated two years prior by a modified radical mastectomy at another hospital. Postoperative histopathological examination (confirmed by the department of pathology of this institute) revealed an original grade II invasive ductal carcinoma(Figure 2A) with a size of 5.0 × 3.0 × 3.0 cm. The axillary lymph node were 1/16 positive. Immunohistochemical stain of the cancer cells was negative for estradiol, progesterone receptors, and positive for C-erbB-2. According to the classification of TNM, the disease was stage IIB(T_2_N_1_M_0_). Due to the poor compliance, the patient only accepted two cycles of chemotherapy after the right modified radical mastectomy (CEF: cyclophosphamide 500 mg/m^2 ^day 1, epirubicin60 mg/m^2 ^day 1 and fluorouracil 500 mg/m^2 ^day 1), and refused any other adjuvant therapies.

**Figure 3:**
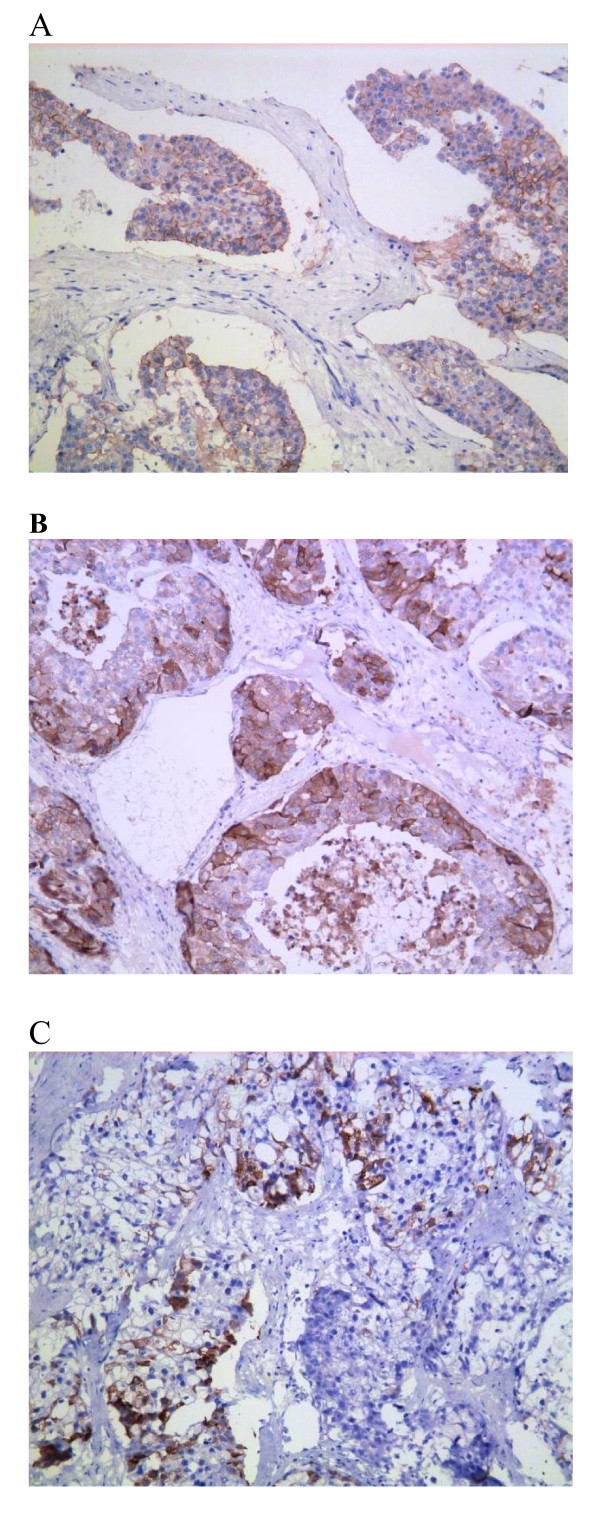
**Immuno - staining of the adrenal metastatic disease (IHC, 20 ×)**. The tumor cells show positivity for C-erbB2 (3A), GCDFP-15 (3B) and Mammaglobin (3C).

On further examination, patient's arterial blood pressure was found normal, as were laboratory measurements including tumor mark CA15-3 (21 U/ml; normal range: 0.0 U/ml-28.0 U/ml). The plasma ACTH was within a normal range (at 0800 h 8.2 pmol/liter; normal range: 1.1 pmol/liter-11.0 pmol/liter; at 1600 h 4.0 pmol/liter, normal range: 0.5 pmol/liter-5.5 pmol/liter). Extensive imaging evaluations including cranial magnetic resonance imaging(MRI), thoracic CT scan, pelvic ultrasonography, and isotope bone scanning (ECT) revealed an isolated disease in the left adrenal gland. We suspected a relapse of the disease. Left adrenalectomy was performed in September 2006. Histopathological examinations confirmed a metastasis event from IDC of the breast as same characteristics of the tumor cells were observed (Figure 2B, C). Immunohistochemical staining on metastasized adrenal tumor showed negative for estradiol, progesterone receptors and P53, but positive for C-erbB-2 (Figure 3A), gross cystic disease fluid protein-15 (GCDFP-15) (Figure 3B) and mammaglobin (Figure 3C). E-cadherin and CK were also positive. After the adrenalectomy, no further adjuvant therapies were performed due to the patient's refusal. The patient is currently in good condition and being followed up at the outpatient clinic without further evidence of recurrence. She has survived for more than three years since the left adrenalectomy for isolated adrenal metastasis from IDC of her right breast.

## Discussion

Metastasis to the adrenal glands is a frequent finding at autopsy and most commonly occurs in patients with lung, gastrointestinal carcinomas and renal [5-9]. Adrenal metastasis from IDC of the breast is relatively rare. A sporadic, isolated, metachronous adrenal metastasis from IDC of the breast is even rarer. Lam KY [9] collected 464 cases with adrenal metastases from various primary tumours during 30 years. The Lungs were the most common primary tumor site (35.4%), followed by the stomach (14.3%), the oesophagus (12.1%) and the liver/bile ducts (10.7%). In this study, breast cancer was the primary site in only 2.9% of cases, however, none of which were solitary adrenal metastasis from IDC. On a review of published work (online PubMed search) till October 2009, we found no report similar to this situation. We believe the patient in our case is the first presentation of a solitary adrenal metastasis from IDC of the breast with a long-term survival description.

Adrenal metastases are often asymptomatic, patients may present adrenal insufficiency if most of the adrenal gland is replaced or destroyed [10]. In addition, cases of adrenal hemorrhage have been reported. For example, Hiroi N [11] described a 56-year-old man who presented with massive retroperitoneal hemorrhage due to adrenal gland metastasis from adenocarcinoma of the lungs. Metastatic tumors are often misdiagnosed as primary adrenal tumors. CT scan and MRI are suitable methods for distinguishing between a metastatic and primary adrenal tumor [12]. Additionally, an F-18 FDG PET/CT scan has also been reported to successfully identify adrenal metastasis [13]. However, although these imaging techniques are helpful in differentiating metastasis from a primary adrenal tumor, the specificity of these imagine-based detection has been always an issue. The final diagnosis should depend on fine-needle aspiration biopsy or metastasectomy. Mammaglobin and GCDFP-15 are two breast-specific antigens that are accepted markers for epithelia of breast origin [14], and are now commonly used to help diagnose metastatic tumors from breast carcinoma. Takeda Y [14] reported that of 20 cases of metastatic breast carcinoma reaching the lungs, 10 (50.0%) were immunoreactive for mammaglobin and 9 (45.0%) for GCDFP-15 in the metastatic tumors. In our reported case the patient was asymptomatic with no abdominal pain or adrenal insufficiency, but an adrenal lesion was indentified by abdominal ultrasonography and CT scan during a follow-up visit. The CT features of the solid mass (size, 5.4 × 7.0 cm) indicated that it was a malignancy disease, which didn't appear as a typical adrenal carcinoma or pheochromocytoma (they usually associated with central necrosis or hemorrhage or calcification). Incorporating the patient's medical history, an adrenal metastasis from breast cancer was concluded. The pathological characteristics of adrenal section finally confirmed this diagnosis, and immunoreactivity for both mammaglobin and GCDFP-15 further supported the finding that the tumor was of breast origin.

Currently, there are no guidelines for treating patients with solitary adrenal metastasis. Studies in lung cancer [5], colorectal carcinoma [6], gastric cancer [7]and renal carcinoma[8] have demonstrated that adrenalectomy for solitary adrenal metastasis is feasible, and could lead to a longer survival in some patients[6,7]. Recent years, with the progressive validation of laparoscopic oncologic surgery in different fields, several authors[4,15,16] have also advocated laparoscopic adrenalectomy(LA) for patients with solitary adrenal metastasis. Compared with open adrenalectomy, LA achieved similar outcomes but with less morbidity and a shorter hospital stay, though this approach may be limited by the size of the metastasis. For a lesion smaller than 4.5 cm, survival is equivalent between the two treatments [15]. As for the predictive factors of survival after adrenalectomy, so far there has been no consistent conclusion. In some studies[5,17], it appears that a disease-free interval (DFI: the time from diagnosis of the primary tumor to the detection of adrenalmetastasis) of >6 months and complete resection are good prognosis factors. However, these were not confirmed in other case series [15,16]. In short, for some selected patients with solitary adrenal metastases, metastasectomy can provide a survival benefit. The 5-year survival rate is approximate 24%~33%[5,8,15-17], but the prognostic factors after the adrenalectomy are still obscure. In our case, the patient had a solitary adrenal gland metastasis two years after the right modified radical mastectomy(DFI = 24 months). With the benefit of the left adrenalectomy, she has lived more than three years without any evidence of recurrence. Thus we suggest that removing all the neoplastic bulk could be curative for some selected patients whose DFI is more than 6 months.

Invasive ductal carcinoma of the breast is considered a systemic disease, the high rate of relapse underlines the need for an effective systemic therapy. Multiple studies have demonstrated that adjuvant therapy for early-stage or advanced breast cancer produces a 23% or greater improvement in disease-free survival and a 15% or greater increase in overall survival rates. Recommendations for the use of adjuvant therapy are based on the individual patient's risk and the balance between absolute benefit and toxicity. Anthracycline-based regimens are preferred, and the addition of taxanes increases the survival rate in patients with lymph node-positive disease[18]. European oncologists tend to prefer FEC-100 than switch to a taxane plus trastuzumab for symptomatic, visceral, metastatic disease overexpressing HER2 [19]. Endocrine treatment option for advanced breast cancer patients with hormone receptor-positive is also a better choice. In this particular case, chemotherapy and trastuzumab treatments were not given because the patients refused further treatment. For an advanced breast cancer, the lack of systemic treatment may affect patient's overall survival and further clinical research is warranted.

## Conclusion

In summary, this is the first reported case of a solitary adrenal metastasis from IDC of the breast with a detailed survival description. For patients in this condition, we suggest that early recognition and adrenalectomy will probably lead to survival benefit. Apparently, this recommendation is based on a rare case and further clinical research is needed.

## Consent

Written informed consent was obtained from the patient for publication of this case report and any accompanying images. A copy of the written consent is available for review by the Editor-in-Chief of this journal.

## Competing interests

The authors declare that they have no competing interests.

## Authors' contributions

XJL and PS conceived concept, participated in drafting of the manuscript and critical review, they also took part in the care of the patient. XFW and FFS assembled data and participated in writing the manuscript. KS carried out the histopathological evaluation and reviewed pathology. All authors read and approved the final manuscript.
